# Characterization of the flexibility of the peripheral stalk of prokaryotic rotary A‐ATPases by atomistic simulations

**DOI:** 10.1002/prot.25066

**Published:** 2016-06-01

**Authors:** Kostas Papachristos, Stephen P. Muench, Emanuele Paci

**Affiliations:** ^1^ Astbury Centre for Structural Molecular BiologyUniversity of LeedsLeedsEngland; ^2^ School of Molecular and Cellular BiologyUniversity of LeedsLeedsEngland; ^3^ School of Biomedical SciencesUniversity of LeedsLeedsEngland

**Keywords:** molecular dynamics, protein mechanics, rotary ATPases

## Abstract

Rotary ATPases are involved in numerous physiological processes, with the three distinct types (F/A/V‐ATPases) sharing functional properties and structural features. The basic mechanism involves the counter rotation of two motors, a soluble ATP hydrolyzing/synthesizing domain and a membrane‐embedded ion pump connected through a central rotor axle and a stator complex. Within the A/V‐ATPase family conformational flexibility of the EG stators has been shown to accommodate catalytic cycling and is considered to be important to function. For the A‐ATPase three EG structures have been reported, thought to represent conformational states of the stator during different stages of rotary catalysis. Here we use long, detailed atomistic simulations to show that those structures are conformers explored through thermal fluctuations, but do not represent highly populated states of the EG stator in solution. We show that the coiled coil tail domain has a high persistence length (∼100 nm), but retains the ability to adapt to different conformational states through the presence of two hinge regions. Moreover, the stator network of the related V‐ATPase has been suggested to adapt to subunit interactions in the collar region in addition to the nucleotide occupancy of the catalytic domain. The MD simulations reported here, reinforce this observation showing that the EG stators have enough flexibility to adapt to significantly different structural re‐arrangements and accommodate structural changes in the catalytic domain whilst resisting the large torque generated by catalytic cycling. These results are important to understand the role the stators play in the rotary‐ATPase mechanism. Proteins 2016; 84:1203–1212. © 2016 The Authors. Proteins: Structure, Function, and Bioinformatics Published by Wiley Periodicals, Inc.

## INTRODUCTION

The ion‐pumping rotary ATPase family consists of three evolutionary related biomolecular nanomachines that share a rotary mechanism but have different functions.^1^ The best characterized of these, the mitochondrial ATP synthase (F‐ATPase), is responsible for catalyzing the biosynthesis of ATP in mitochondria and chloroplasts harnessing the electrochemical potential gradient across biomembranes. The vacuolar ATPase (V‐ATPase), works in the reverse direction, by generating a proton gradient through ATP hydrolysis.^2^ The third member of the family, the archeal ATPase (A‐ATPase) can work in a bi‐directional manner by both utilizing a membrane potential to generate ATP and to pump ions over the membrane using ATP.

Common to all family members is the presence and coupling of two rotary motors, a hexameric soluble ATP synthesis/hydrolysis motor (*F*_1_/*V*_1_/*A*_1_) and a membrane bound domain (*F*_o_/*V*_o_/*A*_o_) responsible for ion movement across the membrane. The motor domain shows strong sequence and structural similarity across the different family members.^1^ In contrast, the composition of the membrane domain depends on organism and localization; it is involved in ion translocation through the membrane, either harvesting the electrochemical gradient across membrane bilayers to synthesize ATP or pumping ions to maintain the ion‐concentration gradient. The two motors are coupled through the rotor and stator connections. It has been suggested that high efficiency of the rotary ATPases can be reconciled with the existence of symmetry mismatch between the two motors, whereby the three‐fold symmetric *F*_1_/*V*_1_/*A*_1_, domain is coupled to the *F*_o_/*V*_o_/*A*_o_ domain which adopts different stoichiometry.^3^, ^4^, ^5^ The presence of elastic energy buffering elements results in a smoothened free energy landscape; assuming that central rotor subunits are torsionally flexible, it has been suggested that “kinetic efficiency” is enhanced.^6^ Recently there has been a greater understanding of the inherent flexibility within the ATPase family, with electron microscopy (EM) studies,^7^, ^8^ elastic network model (ENM) normal‐mode analysis (NMA),^7^, ^9^ and ion mobility mass spectrometry investigations,^10^ all highlighting the presence of conformational flexibility within rotary A/V‐ATPases.

**Figure 1:**
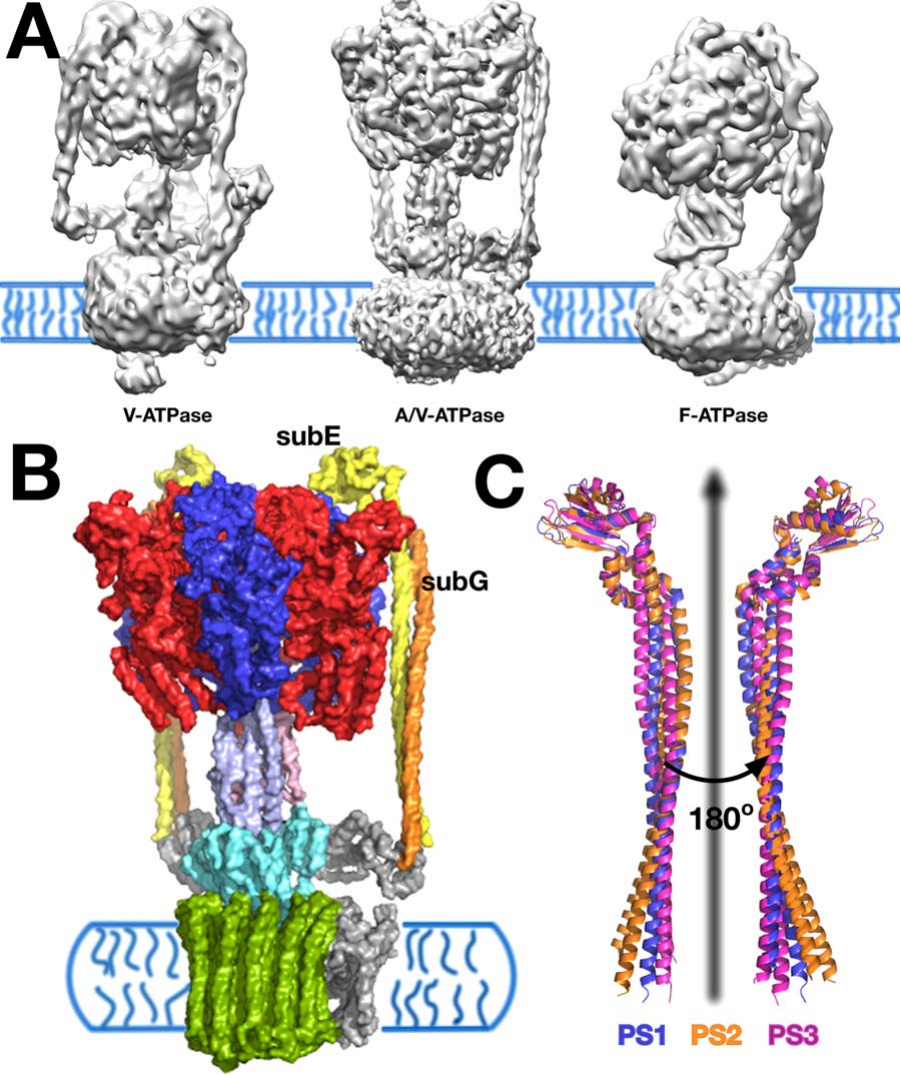
Structural organization of the prokaryotic A‐ATPase. (A) Electron‐microscopy 3D maps of ion‐pumping rotary ATPases: V‐ATPase (left), A/V‐ATPase (middle) and F‐ATPase (right). (B) Two copies of the peripheral stator EG complex (colored in yellow and orange) connect the catalytic subunits A/B (subunits colored in blue and red) with the membrane‐bound subunit I (colored in grey). (C) Superposition of the three peripheral stator EG conformers PS1, PS2 and PS3.

An important distinctive feature of the different rotary ATPase types is the number of peripheral stator stalks that mechanically connect the catalytic subunits of the soluble motor to the membrane sector, with the F‐ATPase containing one, the A‐ATPase two and the V‐ATPase three stators.^1^ X‐ray crystallography has provided insights into the structure of the prokaryotic A‐type peripheral stator EG complex from *Thermus thermophilus* (*T. thermophilus*) and the corresponding yeast V‐type EG complex.^9^, ^11^, ^12^ Both the A‐ and V‐type complexes share the same overall structural organization: the two constituent subunits of the stator are bound to form a C‐termini globular head domain that interacts with the catalytic A/B subunits of A_1_/V_1_‐ATPase and a N‐termini right‐handed coiled coil (rhcc) tail domain interacting with the peripheral collar subunits (Fig. 1).

Crystal structures of the V‐type EG stator has revealed a unique, disordered section within the coiled coil which could contribute to the S‐shaped conformations of the V‐type stators as revealed in EM reconstructions of intact V‐ATPases.^8^, ^12^, ^13^ This feature is not present within the A‐ATPase with a significantly straighter conformation seen in the EM reconstructions.^14^ Conformational dynamics and mechanics of the EG stators are expected to have a great impact on the overall conformational flexibility of the intact rotary ATPases and possible mechanical differences between the A‐ and V‐type stators could correlate with differences in stoichiometry and conformations of bound EG stators. Based on two crystal structures of the *T. thermophilus* A‐ATPase peripheral stalk, Stock *et al*.^9^ have reported that catalytic subunits A_3_B_3_ of the A‐ATPase wobble during the rotary catalysis cycle with the peripheral stators accommodating the global conformational changes associated with ATP turnover, as a result of their inherent flexibility. This flexibility has also been seen directly with EM within the related V‐ATPase with the EG stator elements predicted to be important for this flexibility.^8^ More recently, the yeast V‐ATPase has been resolved in three distinct conformational states with the EG stators showing a clear difference in their conformation during catalytic cycling.^8^ Understanding the conformational flexibility of the EG stators which couple the two motors in A‐ and V‐ATPases is essential to dissecting the origin of rotary ATPases' mechanics and its relevance to function.

In the current work, we employed atomistic molecular dynamics (MD) simulations of the isolated EG stator to study its intrinsic conformational flexibility in solution. Because of its specific structural organization and the right‐handed coiled coil mechanics, the prokaryotic A‐ATPase EG stator preserves its structural integrity while exhibiting global shape fluctuations that would allow large‐scale, tertiary conformational changes to take place. Rather than sampling defined states the stator explores a broad free energy basin; the most recently solved structure (referred here to as PS2 following previously introduced nomenclature^9^) represents a strained microstate that is only marginally populated in solution at near ambient conditions. The apparent persistence length of the right‐handed coiled coil domain calculated from equilibrium simulations is of the same order of magnitude as for other common coiled coil domains. However, the elastic properties are highly heterogeneous, and differ from those of an elastic rod.

## METHODS

Three conformers of *T. thermophilus* A‐ATPase EG stator (PS1, PS2, and PS3) were used as initial structures for molecular‐dynamics (MD) simulations. The first structure (PS1, PDB ID: 3K5B^11^) comprises residues 21–120 from subunit G and residues 3–188 from subunit E at 3.1‐Å resolution. Missing residues in subunit E (143,144) were modeled with MODELLER.^15^ The second crystal structure (PS2, PDB ID: 3V6I^9^) was determined at 2.25 Å resolution; it exhibits a bending and twisting deformation with respect to PS1.^16^ The third conformer (PS3) is a structural model^9^ generated from flexibly fitting the EG crystal structure into the 3D EM reconstruction from the intact A‐ATPase.^17^ Three simulations have been performed for the wild‐type (WT) sequence starting from each of the conformers PS1, PS2, and PS3, with the MUTATE plugin of VMD^18^ being used to mutate selenomethionine in PS1 to leucine as in the wild‐type sequence. Summary of all the simulations performed is reported in Table 1.

**Table 1:** Summary of the Simulations Carried out in This Study

Trajectory	Initial structure	Time (ns)	Trajectory ID
1	PS1	250	sim1
2	PS2	140	sim2
3	PS3	170	sim3
4	—	230	sim4

Trajectory 4 (sim4) is the union of trajectory 2 (sim2) (40–140 ns) and trajectory 3 (sim3) (40–170 ns) considered to best represent the equilibrium population of the wild‐type stator in solution.

All initial structures were immersed in an orthorhombic water box; solvent molecules <2.5 Å from any protein atom were deleted. Sodium and chloride ions were added so that the solution is neutral and the ionic strength close to physiological (∼140 m*M*, resulting in 37 sodium ions and 32 chlorine atoms for 24,256 water molecules in the simulation cell). Molecular dynamics simulations were performed with NAMD2.8.^19^ The CHARMM22 force field with the CMAP correction^20^ was used for the protein, while the modified TIP3P^21^, ^22^ model was used for water. Periodic boundary conditions were applied and long‐range electrostatics were calculated with particle‐mesh Ewald^23^ and grid spacing <1 Å. A cut‐off distance of 12 Å was used to truncate the van der Waals interactions with a switching function between 10 and 12 Å. The system was initially energy minimized with 10,000 conjugate‐gradient steps. Positional restraints were applied on the backbone atoms of the protein with a force constant of 50 kcal mol^−1^ Å^−2^. Following this, the system was heated from 0 to 310 K using velocity rescaling, after which the temperature was maintained at 310 K while positional restraints were progressively switched off. Simulations were performed in the NPT thermodynamic ensemble. Langevin dynamics^24^ with a damping constant of 1 ps^−1^ was employed to maintain a temperature of 310 K. Pressure control was achieved with a Langevin piston barostat^25^ at 1 bar with a period of 200 fs and a decay rate of 100 fs. All covalent bonds involving hydrogens were constrained with the RATTLE algorithm.^26^ An integration time‐step of 2 fs was used; short‐range interactions have been evaluated at every time step and long‐range electrostatic interactions every second time step.

Average structures were calculated after aligning the structures to the initial conformation (by minimizing the root‐mean‐square deviation of the Cα atoms positions). The covariance matrix $C_{ij}$ of coordinates $x_{i}$ and $x_{j}$ was calculated and diagonalized. The resulting eigenvectors or principal modes were sorted by decreasing eigenvalue. Trajectories were projected onto the first three eigenvectors to obtain the PC coordinates $q_{i}$. PCA calculations were performed with Wordom,^27^ while calculations bond lengths, bond angles and dihedral with VMD1.9.^18^

## RESULTS AND DISCUSSION

**Figure 2:**
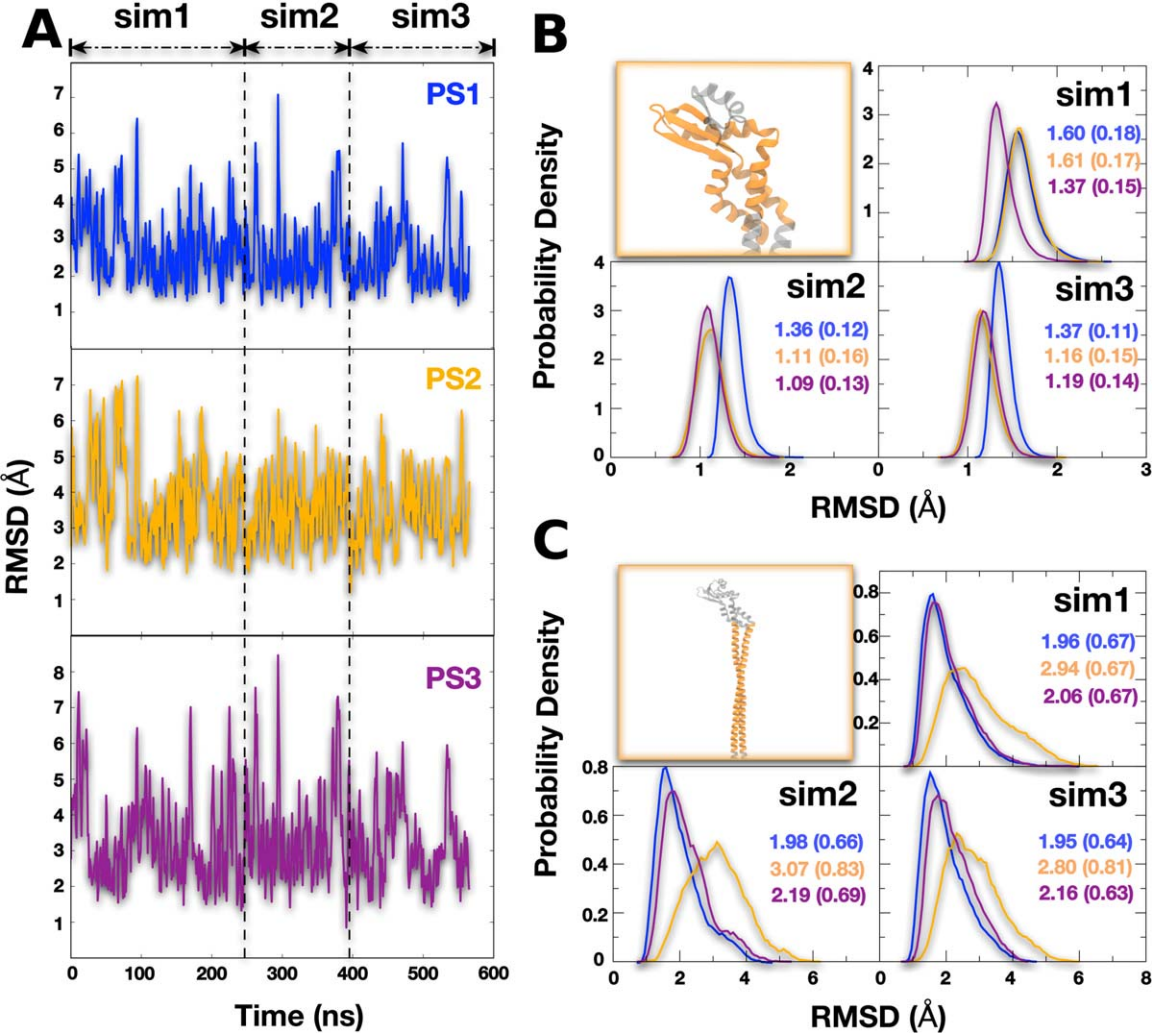
(A) Time series of the RMSD for all MD trajectories (sim1, sim2, and sim3) of the EG complex with PS1 (blue), PS2 (orange), and PS3 (purple) as reference structures. Dashed vertical lines mark the boundaries between independent trajectories sim1‐sim3. (B) Distributions of RMSD for the head domain for all simulations from the three different experimental structures; the head domain in structures PS2 and PS3 is similar and trajectories started from these structures appear to be mutually similar. (C) Distributions of RMSD for the tail domain for all simulations from the three different experimental structures; all three trajectories appear similar, and all deviate most from PS2 which has a kink in the tail domain. [Color figure can be viewed in the online issue, which is available at wileyonlinelibrary.com.]

The root‐mean‐square deviation (RMSD) of the stator relative to the reference structures PS1/PS2/PS3 is shown in Figure 2(A). Residues subE:123–150, which are part of the head domain, form a disordered loop in PS1 and a β strand in PS2 and PS3, were not included in the RMSD calculation. The total RMSD shows that each structure may deviate considerably from the initial structure (up to 8 Å) but overall the RMSD is stable and small considering the size of the EG stator.

To eliminate the effect of interdomain motions, RMSD values for the head and tail domains were calculated [shown in Fig. 2(B,C)] for all independent trajectories using PS1/PS2/PS3 as reference structures. The head‐domain conformation remains close to the reference structures of PS2 and PS3 but deviates more from PS1 due to local structural differences of subunit E in the region 123–150 affecting head‐domain dynamics. The RMSD for the tail alone in contrast shows that PS2 deviates the most from the conformations sampled during the simulations. The main difference between the reference structures of PS1 and PS3 compared to PS2 is that in the latter the tailed‐domain coiled‐coil is bent in an apparently strained conformation.

**Figure 3:**
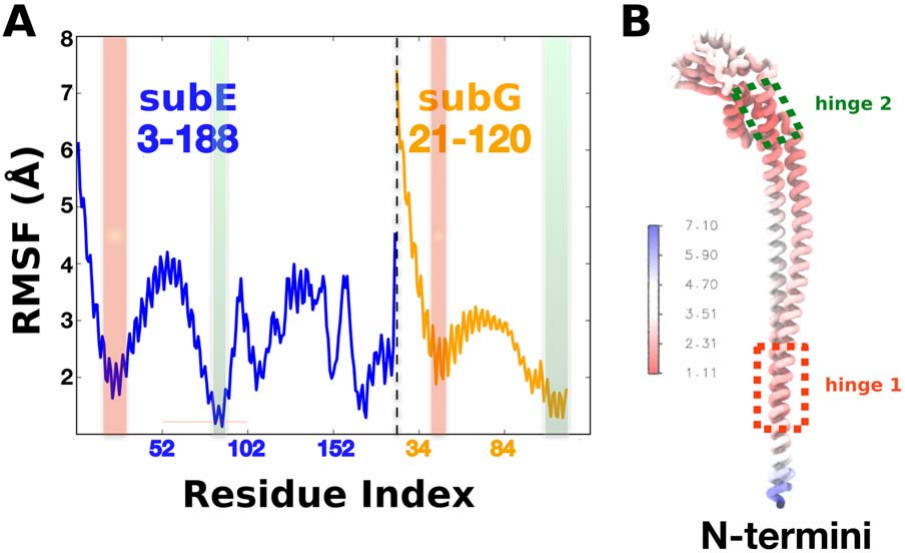
(A) RMSF for the whole peripheral stalk complex. Hinge residues are color‐shaded (hinge 1, red and hinge 2, green). (B) The average structure colored according to RMSF values. Hinge regions are highlighted in dashed rectangular boxes. Local changes around hinge 2 allow level‐arm movements of the coiled‐coil domain translated into large‐scale movements of the N‐terminal end. [Color figure can be viewed in the online issue, which is available at wileyonlinelibrary.com.]

The root mean square fluctuations (RMSF) around the average structure are shown in Figure 3. The average structure has been computed on trajectory sim4, which comprises of both trajectory sim2 (40–140 ns) and trajectory sim3 (40–170 ns) and best represent the equilibrium population of the wild‐type stator in solution. The RMSF profile indicates the presence of two relatively rigid regions connected by hinge regions of low mobility. The first hinge comprises residues subE:19–31 and subG:44–51 near the N‐termini of subunits E and G where the peripheral stalk connects to the soluble domain of the membrane subunit I [Fig. 3(B)]. The second hinge consists approximately of residues subE:81–88 and subG:107–120 forming a “neck” region at the boundary of the globular head and the right‐handed coiled coil [Fig. 3(B)]. These hinge regions allow the EG stator to preserve its structural stability and rigidity whilst allowing the stator to accommodate tertiary displacement of A_3_B_3_ sub‐complex relatively to the membrane sector as suggested in Ref. 9. The rod‐like shape of the tail suggests a lever‐arm movement where local changes near the second hinge are translated into large changes at the N‐termini end of the EG stator.

Simulations started from PS2 and PS3 tend to the same relaxed state in solution. Secondary structure differences in the head domain between PS1 and PS2/PS3 affect the head dynamics and are associated with differences in apparent flexibility of the coiled coil N‐terminal end. However, in all cases, except PS1 and regardless of the degree of flexibility, the intact EG stator preserves an average structure that has a straight coiled‐coil tail. The latter contrasts with short MD simulations of the A_3_B_3_E_2_G_2_ 40^+^ ion in gas phase, performed to investigate structural dynamics under ion mobility spectrometry (IMS) conditions and interpret collisional cross section (CCS) measurements.^10^ However, it's important to note that those simulations were carried out over a short time (a few nanoseconds) without solvent and in a highly charged state to mimic the gas phase; simulations presented here include solvent effects and have been carried over about half a microsecond.

### Principal component analysis

Principal component analysis (PCA) was performed on trajectory 4. Eigendecomposition of the covariance matrix of conformational fluctuations around the average structure resulted into the principal modes of motion that best represent the conformational variability observed in a simulation between conformations. The first three PC modes account for ∼80% of total conformational variability suggesting they might be relevant for the function.

**Figure 4:**
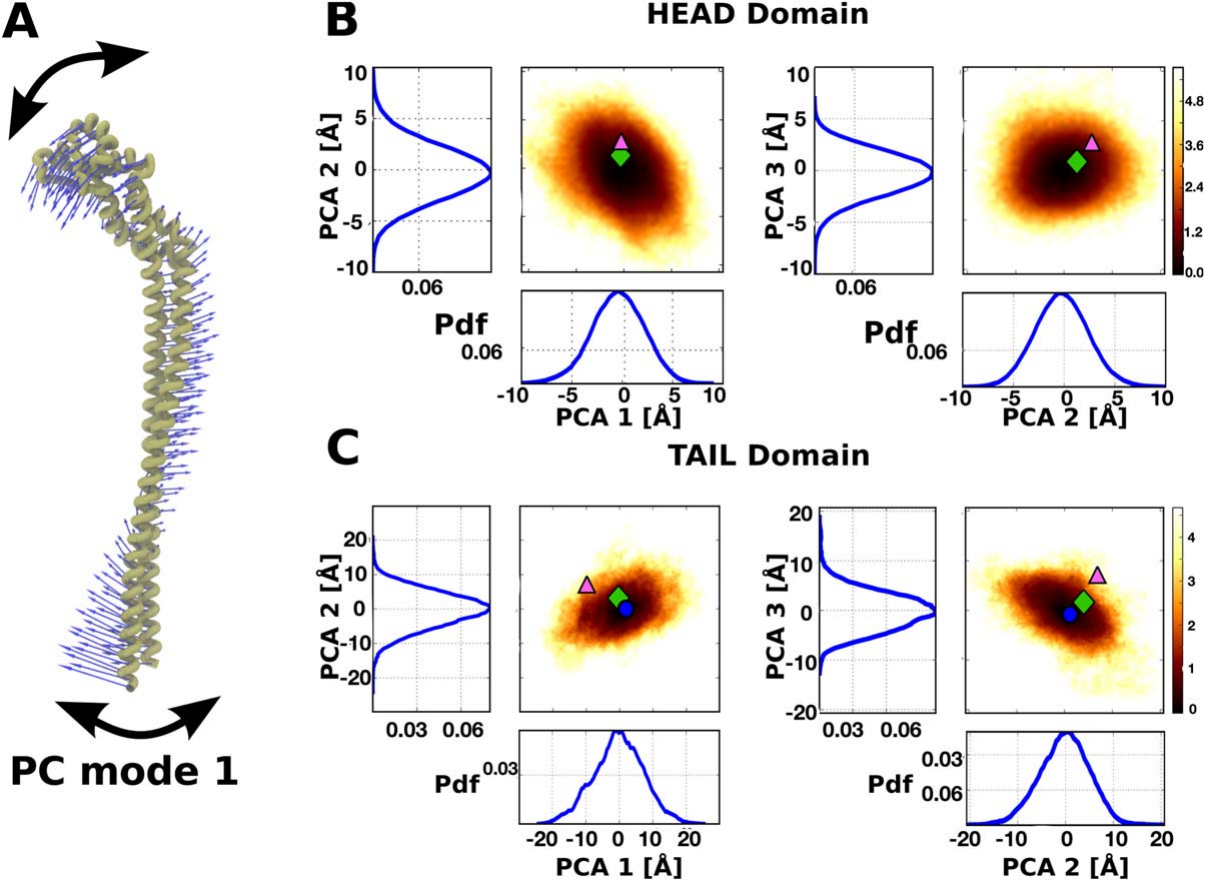
Principal component analysis of the dynamics of the EG stator. (A) “Porcupine” representation of the first eigenvector. Projection of the trajectory 4 (sim4) on the subspace spanned by the first three eigenvectors for the head (B) and coiled coil tail (C) domains in isolation. Reference structures PS1, PS2, and PS3 are shown as circle (blue), triangle (pink) and rhomboid (green) symbols, respectively. PS1 has been omitted in (B) due to the differences in local secondary structure and associated dynamics (see main text for details). [Color figure can be viewed in the online issue, which is available at wileyonlinelibrary.com.]

The first PC eigenvector from analysis of the whole stator corresponds to a bending motion where the head and tail domains of the EG stator permit cooperative wobbling motions of the *A*_1_ and *A*_o_ domains [Fig. 4(A)].

Modes 2 and 3 share some similarity with mode 1 but more complex motions are observed such as twisting within the tail domain. To remove inter‐domain motions and probe the flexibility within each domain individually, PCA was carried out separately for the two head and tail domains. In Figure 4(B), the PMF along principal components 1 and 2 or 2 and 3 are shown for the head domain. Reference structures PS2 (triangle) and PS3 (rhomboid) are also projected onto the PMF to show the stability differences between the two conformations. In Figure 4(C), corresponding results are shown for the tail domain along with the three reference structures PS1 (circle), PS2 and PS3. PC analysis suggests that the straight tail of PS1/PS3 is the most likely conformation in solution conditions, while the PS2 conformer adopts a higher‐energy bent arrangement within its tail domain.

In summary, PC analysis suggests that structures PS1, PS2, and PS3 are not clearly identifiable as stable or metastable states but accessible conformational microstates in ambient conditions. Moreover, conformations with the tail bent as in PS2 are marginally populated relative to PS1 and PS3. It has been suggested^9^ that the structures of PS1, PS2, and PS3 represent states of the EG stator within the holo‐complex that exist to accommodate the conformational change associated with the catalytic cycle.^9^ Our results suggest that the bent conformation of the tail region as found in PS2 is a higher‐energy, strained conformation that spontaneously converts into the lower energy conformation of PS1, unless constraints imposed by a crystal lattice or the subunit interactions it makes within the rotary ATPase stabilize it.

### Coarse‐grain structural analysis

**Figure 5:**
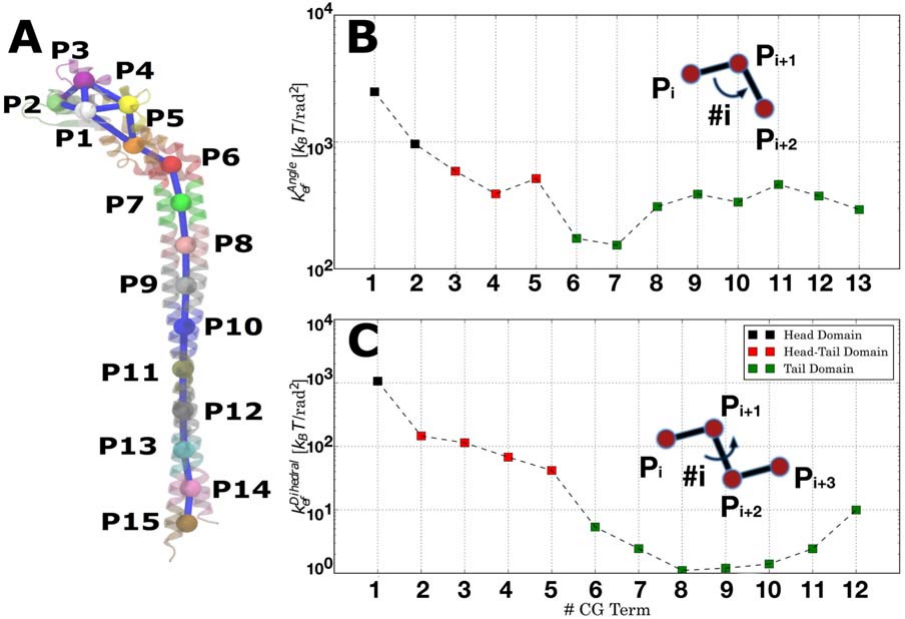
Coarse‐grain analysis of EG‐complex flexibility. The peripheral stator complex is divided into subdomains represented by coarse‐grain beads (A). Conformational flexibility is quantified with effective elastic spring constants of angles and dihedrals defined by consecutive coarse‐grain beads from P1 to P13 for angles (B) or from P1 to P12 for dihedrals (C) (axis y plotted on logarithmic scale). Domain‐specific angle and dihedral elastic spring constants are shown in black and green color for the head and tail domain respectively. Terms that involve the P5 bead that connects the two domains are shown in blue color and referred to as “neck‐domain” terms. Definitions of the coarse‐grain domains used for the analysis are given in Table II. [Color figure can be viewed in the online issue, which is available at wileyonlinelibrary.com.]

To gain further insight in conformational flexibility of the complex, local coarse‐grain geometric analysis was carried out. To this end, the EG complex was divided into fifteen subdomains by mapping subsets of residues in spatial proximity onto beads that capture the overall topology of the complex in a manner previously described^28–31^ with each GC bead representing 300 atoms on average [Fig. 5(A)]. Four beads (P1 to P4) were assigned to the head domain and ten beads (P6 to P15) to the right‐handed coiled‐coil domain. Bead P5 resides between the head and tail domains and will be referred to as the “neck domains” (see Table 2 for detailed definition of all CG beads).

**Table 2:** Coarse‐grain (CG) Representation of the Peripheral Stalk

# CG bead	Subunit E residues	Subunit G residues
P1	122–128, 143–153, 165–166	—
P2	110–120, 154–164	—
P3	105, 108–109, 121, 129–142	—
P4	92–104, 106–107	120
P5	84–91, 167–178	112–119
P6	79–83, 179–188	102–111
P7	69–78	93–101
P8	61–68	84–92
P9	52–60	74–83
P10	42–51	65–73
P11	34–41	56–64
P12	24–33	47–55
P13	16–23	38–46
P14	6–13	32–37
P15	3–5	21–31

Definition of CG domains in terms of residue IDs that belong to each domain.

Effective elastic spring constants were defined for all angles and dihedrals defined by consecutive beads according to $k_{i}=\frac{k_{B}T}{2\sigma_{i}^{2}}$ where $k_{B}$ is the Boltzmann constant, *T* the temperature of atomistic simulations and ${\sigma^{2}}_{i}$ the sample variance of the corresponding internal coordinate. For example, angle #1 is defined by beads P1, P2, and P3. Similarly dihedral #1 is defined by beads P1, P2, P3, and P4. Effective elastic spring constants are shown in Figure 5 for 13 angles and 12 dihedrals. Those “elasticity profiles” reveal local bending and torsional flexibility heterogeneity across the complex. In particular, significant decrease in bending stiffness is observed from the head domain (P1 bead) to the tail domain (P6 domain) via the neck domain. Most importantly, there are large differences in bending flexibility within the tail domain with the segment spanned by P6–P9 beads characterized by enhanced flexibility compared to the rest of the tail domain. Regarding the torsional flexibility profile [Fig. 5(B)], the tail domain is significantly more flexible than the head and neck segments and heterogeneous with torsional elastic constant adopting minimum value within the segment spanned by P9–P12 beads.

### Right‐handed coiled‐coil tail persistence length

Coiled‐coil is a ubiquitous structural motif consisting of two or more α helices wrapped around each other. Tight packing of hydrophobic amino acids side chains at the core of the coiled‐coil in a “knobs‐in‐holes” fashion results in mechanical stability and rigidity. Various experimental and theoretical studies examined dynamical and mechanical properties of coiled coil domains of structural proteins and linear molecular motors (steppers). Single‐molecule force spectroscopy has been employed to probe the force‐extension diagram of myosin coiled coils^32^ while electron microscopy and single particle analysis of 2D images are well suited for characterization of equilibrium thermal fluctuations of large coiled‐coil proteins such as tropomyosin.^33^ A property usually reported is the apparent persistence length as a measure of protein stiffness as it is related to the decay of spatial correlations along the contour length of the polymer.

The tail domain of the EG stator shares characteristic features with other known coiled‐coil dimers. Specifically, the tail domains are right‐handed with a combination of two different pitches and it is unclear whether they behave similarly to their left‐handed counterparts. To our knowledge, there are no measurements of the persistence length of the EG stator in the A/V‐ATPase family. However, Junge *et al*.^34^ have estimated torsional and lateral elasticity for the analogous b_2_ complex from *E. coli F*_o_ *F*_1_‐ATPase, from which a persistence length *L*_p_ of ∼710 nm could be estimated. Hence the *b*_2_ complex appears to be much stiffer than other coiled coils; for example a persistence length *L*_p_ = 25 nm has been reported^32^ for myosin II.

From the fluctuations of the distance between the two ends of the EG stator we estimated the persistence length using the formula:

(1) $$〈R_{ee}^{2}〉=2LL_{p}-2{L_{p}}^{2}\left[1-\exp\left(-\frac{L}{L_{p}}\right)\right]$$

This expression holds for a worm‐like chain, where $R_{ee}$ is the end‐to‐end distance, L the contour length, and $L_{p}$ the persistence length.

Numerical solution of Eq. (1) gave a value of *L*_p_ = 105 ± 42 nm, comparable to estimations of conventional coiled‐coil domains (*L*_p_ = 25–160 nm), e.g., myosins.^32^, ^35^, ^36^ These results suggest that the tail of the EG peripheral stalk is stiff enough to support its role as a stator, consistent with cryoEM density maps where straight segments corresponding to EG subcomplexes were seen,^37^ but much less stiff than the stator of *E. coli* F_O_F_1_ ATP synthase allowing for global bending motions of intact A/V‐ATPase.

## CONCLUSIONS

Atomistic molecular dynamics simulations of the *T. thermophilus* EG stator in explicitly modeled aqueous solution show that the EG stator is not dominated by a single structure; rather it is best represented by a continuum of structures. Available crystallographic data provides insight into the structural organization of the EG stator and some hint into the conformational flexibility of this important A‐ATPase component. Three conformers have previously been proposed to explain the dynamics associated with catalytic turnover of the rotary A‐ATPase. Two experimental structures, PS2 and PS3^9^ differ mostly in that PS2 has a bent tail domain. Simulations started from either PS2 or PS3 converge rapidly to a single basin on the free energy landscape where local structure is well preserved and the tail is extended on average. The bent conformations of the tail as seen in PS2 are often explored but not populated and may represent a strained conformation, which could be stabilized by interactions in the holo‐complex. The experimental structure PS1^11^ differs from the other structures particularly in the head domain where an unstructured region in PS1 is instead a β sheet in PS2 and PS3. In the simulation started from PS1 the loop does not form a β structure that is conserved in the simulations started from PS2 and PS3.

The persistence length of the EG stator, a right‐handed coiled coil, estimated from our simulations, is about 100 nm, close to that observed for conventional coiled coils.^32^, ^33^, ^35^ An analysis of the all‐atom simulation based on a superimposed coarse‐grain model with a relatively small number of bonds, angles, and dihedrals highlights a behavior different from that of a homogeneous elastic rod. The EG stator flexes around “hinges,” or regions of small positional fluctuations, in the coiled coil domain and in the linkage of the head and coiled coil domains.

The large‐scale deformations within the EG stator observed in gas‐phase molecular dynamic simulations and proposed to explain variability of experimental correlation cross section values from ion mobility mass spectrometry experiments^10^ are not observed in our simulations.^10^ This is likely due to fact that the simulation was performed in gas‐phase to reproduce the conditions of the mass spectrometry experiment. Here we can show that if the effects of solvation are taken into account the EG stator, while flexible, preserves its local structure even in isolation.

Stock *et al*.^9^ have proposed, based on the different conformational states of the EG stator, that during catalysis the A_3_B_3_ axis tilts relative to the membrane by about 7°, a feature also seen for the eukaryotic V‐ATPase in electron microscopy.^7^, ^8^ Combined, thermal conformational fluctuations within the rotor and stator of rotary A‐ATPases would preserve the same ground state in terms of global shape while accommodating any structural rearrangements within the *A*_1_ motor during rotary catalysis. Importantly the V‐ATPase has recently been solved in two distinct catalytic states with the stator network being dominated by the interactions it makes to subunits within the collar region and not nucleotide occupancy.^13^ This has significant implications in our understanding of the stator role and implies that the apparent flexibility is required to allow the same basic building block to adopt significantly different conformations within the structure whilst maintaining the same rigidity to resist torque. Our analysis shows that the coiled coil region maintains the rigidity whilst the hinge regions permit plasticity to fit within the complex. Further work is required to probe in detail any differences in the mechanical properties of peripheral stators among the members of rotary ATPase family (F ATP synthase, A/V, and V‐ATPases). The results presented in this article suggest that available experimental structures are snapshots of possible conformations of the stator but that a full representation of the stator must include dynamics, as it is a well‐defined but highly compliant structure with specific mechanisms that allow it to strain and adapt.
